# Association between out-of-pocket expenditure and health-related quality of life among patients receiving cancer treatment: a cross-sectional study from Nepal

**DOI:** 10.1186/s12955-025-02404-9

**Published:** 2025-07-15

**Authors:** Pratik Khanal, Suman Sapkota, Nirmal Poudel, Achyut Raj Pandey, Bidwata Bhattarai, Etna Khatiwada, Ravi Kant Mishra, Biraj Man Karmacharya, Shiva Raj Adhikari, Kjell Arne Johansson, Krishna Kumar Aryal

**Affiliations:** 1 Bergen Centre for Ethics and Priority Setting in Health (BCEPS), Department of Global Public Health and Primary Care, University of Bergen, Bergen, Norway; 2 Nepal Health Economics Association, Kathmandu, Nepal; 3 Department of Public Health and Community Programs, Kathmandu University School of Medical Sciences, Dhulikhel, Nepal; 4 Central Department of Economics, Tribhuvan University, Kathmandu, Nepal

**Keywords:** Cancer, EQ-5D-5 L, Health-related quality of life, Nepal, Out-of-pocket expenditure, Patient satisfaction

## Abstract

**Background:**

The financial burden of cancer care may significantly impair patients’ health-related quality of life (HRQoL), yet the extent and nature of this relationship remain underexplored, particularly in low-resource settings. This study aimed to report the HRQoL of patients currently receiving treatment for selected cancers (breast, cervical, lung, oesophageal and stomach) in Nepal. We further investigate the association of out-of-pocket expenditure (OOPE) with HRQoL.

**Methods:**

A cross-sectional survey was conducted from April to May 2024 among 353 patients undergoing cancer treatment in two tertiary cancer hospitals in Nepal. We used the European Quality of Life 5 dimensions (EQ-5D-5L) and the European Quality of Life Visual Analogue Scale (EQ-VAS) to obtain their HRQoL. Similarly, we collected sociodemographic and treatment-related data, including OOPE and patient satisfaction. We used the ordinary least squares estimation with robust standard errors to identify the association between OOPE and HRQoL (EQ-5D-5L index score).

**Results:**

The mean (SD) EQ-5D-5L index score was 0.39 (0.42), and the mean (SD) EQ-VAS score was 56.65 (21.71). Anxiety/depression and pain/discomfort were the most common reported problems (> 90% of the study participants), whereas pain/discomfort had the greatest disability weight (0.17). In the regression analysis, the logarithms of OOPE (β = -0.086; 95% CI: -0.132 to -0.040) was significantly associated with a lower EQ-5D-5L index score. Other significant covariates included being currently not married (β = -0.149; 95% CI: -0.274 to -0.024), having stage IV cancer during diagnosis (β = -0.212; 95% CI: -0.364 to -0.061) and patient satisfaction score (β = 0.015; 95% CI: 0.001 to 0.030).

**Conclusions:**

The study revealed a moderate quality of life among patients currently receiving cancer treatment in Nepal. Higher OOPE along with stage IV cancer during diagnosis and being currently not married were associated with lower HRQoL, whereas higher patient satisfaction score was associated with higher HRQoL. These insights might be helpful for providing targeted interventions such as emphasizing early diagnosis and management and focusing on patient satisfaction and those at financial risk to improve the HRQoL of people with cancer.

**Supplementary Information:**

The online version contains supplementary material available at 10.1186/s12955-025-02404-9.

## Introduction

Despite advancements in oncology care in recent decades, 20 million new cancer cases, 9.7 million cancer-related deaths and 253 million disability-adjusted life years (DALYs) were estimated for 2021 globally [1–3]. In addition to reduced survival outcomes, patients with cancer also experience symptom burdens; treatment-related side effects; and emotional, psychological, and financial distress [4]. In low- and lower- middleincome countries (LLMICs), delays in health facility presentation, diagnosis, and treatment further affect patients, leading to poor survival outcomes and a considerable decline in health-related quality of life (HRQoL) [5–9].

Measuring HRQoL through patient-reported outcome measures has been used across several treatment fields including cancer to quantify the impact of disease or treatment on patients’ lives and daily conditions. HRQoL is a multidimensional construct that encompasses physical, emotional, social, and functional well-being, providing a comprehensive assessment of a patient’s overall health status [10]. It serves as a critical metric for evaluating the impact of both disease progression and therapeutic interventions. Most research on measuring patient-reported HRQoL has, however, focused on clinical outcomes, often neglecting the broader psychosocial and economic dimensions of cancer care [11]. High out-of-pocket expenditure (OOPE), loss of income, and limited financial protection exacerbate the financial strain on cancer patients, further diminishing their overall well-being and satisfaction with care [12]. Studies from high-income countries (HICs) have shown negative impact on the HRQoL of patients due to financial strain resulting from cancer treatment [11, 13–15]. However, such studies in LLMICs such as Nepal are lacking, where patients often face additional challenges due to the limited healthcare infrastructure, lack of access to advanced medical treatments, and inadequate financial resources [16–19]. Importantly, few studies have assessed the associations between the subjective financial burden of cancer (e.g., financial toxicity) and HRQoL [14, 20, 21] or between objective financial burden (direct medical and nonmedical costs, OOPE) and HRQoL [15, 22–26]. A 2023 systematic review revealed only four studies (three of which were conducted in HICs and two related to cancer) examining the association between OOPE and HRQoL [27].

In this context, this study aims to report HRQoL among patients undergoing treatment for five specific cancers (lung, breast, cervical, stomach, and oesophageal) in Nepal. We further explore the association of OOPE with HRQoL. The findings are expected to generate evidence to inform policies aimed at reducing the economic burden of cancer care and improving patient-centred outcomes.

## Methods

### Study design and study participants

A cross-sectional hospital-based study was conducted among patients undergoing cancer treatment in two tertiary cancer hospitals in Nepal. The study sites included BP Koirala Memorial Cancer Hospital (BPKMCH) and Bhaktapur Cancer Hospital (BCH) situated in the Chitwan and Bhaktapur districts of Nepal, respectively. We collected data from April 13 to May 12, 2024, and included patients diagnosed with breast, cervical, lung, oesophageal, or stomach cancer as these cancers are the leading causes of DALYs in Nepal.

### Sampling and sample size

We used a purposive sampling technique to recruit the study participants. Any adult participants (> 18 years) on active cancer treatment for five selected cancers were considered eligible for the study. Since this study is a part of a research project that estimates the patient cost of cancer care in Nepal, the sample size calculation was based on estimating the true population mean cancer cost in one sample situation. The minimum required sample size was 68 for each cancer type (340 in total), which was calculated via the formula for cross-sectional survey, n = Z^2^σ^2^/d^2^, where Z is the standard normal variate corresponding to the confidence level (1.96 for 95% confidence), σ (sigma) is the estimated standard deviation of the variable (Nepalese rupees (NPR) 210,000) [28], and d is the desired margin of error (NPR 50,000). We adjusted the sample size for each cancer type based on their incidence and targeted an additional 15% of the sample to adjust for non or incomplete responses. During the data collection process, we interviewed 387 participants, of whom 353 eligible and with complete data were included in the final analysis.

### Study variables

The study variables included the sociodemographic characteristics of the participants and treatment-related variables, including annual OOPE, patient satisfaction, and HRQoL. The HRQoL score was the dependent variable of the study, while all other variables were independent variables. The study variables are presented in Supplementary Table 1.

### Data collection methods

We conducted face‒to-face interviews with the patients using a structured questionnaire in the Nepali language in both outpatient and inpatient settings. Data were collected digitally via the KoBoToolbox platform by seven trained field researchers with medical, nursing, or public health degrees.

### Data collection measures

#### European quality of life

We used the European Quality of Life 5 dimensions (EQ-5D-5L) and the European Quality of Life Visual Analogue Scale (EQ-VAS) to describe the self-reported health status of the patients [29]. The EQ-5D-5L consists of five dimensions which includes mobility, self-care, usual activities, pain/discomfort and anxiety/depression. Each of these dimensions has five levels of response (1-no problems, 2-slight problems, 3-moderate problems, 4-severe problems and 5-extreme problems/unable to). The score of each dimension is used to derive a health state profile (such as 11255) and is assigned a summary index score on the basis of societal preference weights, ranging from < 0 (equivalent to dead or worse than dead) to 1 (the value of full health) [30]. As country value sets were unavailable for Nepal, we applied value sets from neighbouring country India, which is socio-culturally and geographically similar [31]. In the EQ-VAS, patients rate their perceived health from 0 (the worst imaginable health) to 100 (the best imaginable health). We used the officially translated Nepali version of the questionnaire, obtained from the EuroQol working group [32]. Prior to data collection, the tool was pilot tested in both study sites to ensure linguistic clarity and cultural relevance. The EQ-5D-5L has demonstrated excellent psychometric properties and has been used globally across different populations and conditions including patients with cancer [33]. It has previously been used in Nepal to measure HRQoL among patients with hypertension [34], diabetes [35], tuberculosis [36], and spinal cord and acquired brain injuries [37].

#### Annual OOPE

We collected annual direct medical and direct nonmedical patient costs incurred during the treatment of cancer. Direct medical costs included expenses related to consultation; treatment procedures; laboratory and radiological/imaging investigations; and drugs and medical supplies incurred one year before the survey. Similarly, direct nonmedical expenses included expenses incurred for food, accommodation, transport, clothing, and caretakers. Any reimbursement obtained through private insurance was deducted from these costs, whereas expenses incurred through government subsidies were not included. We validated patients’ information on OOPE through probing, verifying with the service cost of the hospital, and checking patient’s medical bill (wherever feasible). The cost data were collected in NPR and converted to United States dollars (USD) (1 USD = 130 NPR).

#### Patient satisfaction

We measured patient satisfaction using the 7-item Short Assessment of Patient Satisfaction (SAPS) questionnaire [38]. The SAPS assesses the core domains of patient satisfaction, which include treatment satisfaction, explanation of treatment results, clinician care, participation in medical decision-making, respect by the clinician, time with the clinician, and satisfaction with hospital/clinic care. Each of the items is scored from 0 to 4, with the sum ranging from 0 (extremely dissatisfied) to 28 (extremely satisfied).

### Data analysis

We analysed the data via STATA version 18 and R version 4.3.0. Descriptive analysis was conducted by calculating frequencies and percentages for categorical variables and means and standard deviations (SD) for continuous variables. For bivariate analysis, we applied the Mann‒Whitney U test and Kruskal‒Wallis H test to identify associations between independent variables and the EQ-5D-5L index score. Similarly, we used the Spearman rank correlation coefficient to assess the strength and direction of correlation between continuous variables (patient satisfaction, OOPE, EQ-5D-5L index score and EQ-VAS score).

We used multiple linear regression models with ordinary least squares estimation and robust standard errors to identify the association of OOPE with EQ-5D-5L index score. This approach is preferred in addressing heteroskedasticity [39]. Variables with p values < 0.2 in the bivariable analysis were included in the regression model. The included variables were age, place of residence, educational status, type of family, marital status, occupational status, type of cancer, cancer stage at the time of diagnosis, admission to inpatient care in the past 365 days, patient satisfaction score, and OOPE. To examine the associations between OOPE and EQ-5D-5L index score, we specified two regression models (Model I and II) with different operationalisations of OOPE but with same covariates. In Model I, OOPE was included as a continuous variable using the natural logarithm (log of OOPE) to account for right-skewness while in Model II, OOPE was categorized into quintiles (lowest to highest) to facilitate interpretation across expenditure levels.

We specified the models in several functional forms to optimize the performance of the linear regression. To maintain model parsimony and achieve a better fit, we conducted an analysis of variance. We assessed multicollinearity via variance inflation factors, with the highest being 1.96, indicating the absence of significant collinearity among the predictors. The output of the regression model is reported in terms of regression coefficients (*ß*), 95% confidence intervals (CIs) of *ß* and p values. We also present the predicted values of the EQ-5D-5L index score, resulting from the regression model, as a function of OOPE and patient satisfaction scores, stratified by cancer stage, in Fig. 2.

### Ethics

Written informed consent was obtained from the study participants. An information sheet containing the study purpose, the risks and benefits of participating in the study, the voluntary nature of participation and the right to withdraw at any time was provided and read aloud to the participants. The interviews were conducted in places where privacy was ensured and patients felt comfortable. The study obtained ethical approval from the ethical review board of the Nepal Health Research Council and an approval waiver from the Regional Committees for Medical and Health Research Ethics, Norway.

## Results

### Health profile of the study participants

**Fig. 1:**
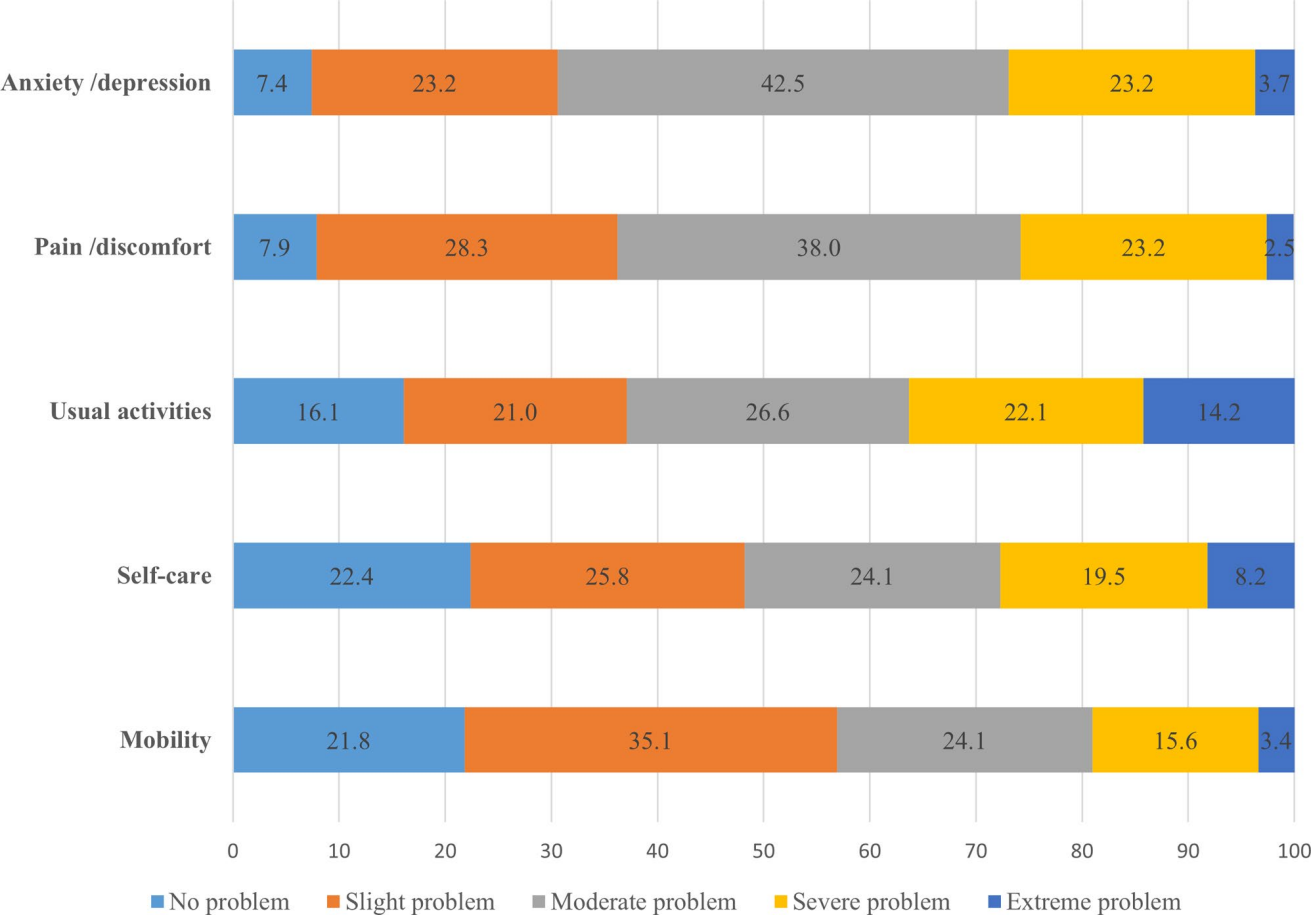
Health profile of the participants based on five dimensions of the EQ-5D-5L

The health state profiles of the participants as per EQ-5D-5L are presented in Fig. 1. Most participants reported moderate to extreme problems with the dimensions of anxiety/depression (69.4%), pain/discomfort (63.7%), usual activities (62.9%), self-care (51.8%) and mobility (43.1%). A few of the participants reported that they had no problems related to anxiety/depression (7.4%) or pain (7.9%). Overall, the proportion of the participants reporting no problems to all the five dimensions was 1.13% (*n* = 4/353) while there was none reporting highest severity across all the dimensions. Across the five dimensions, the mean (SD) disability weight was highest for pain/discomfort, 0.17 (0.15) and lowest for anxiety/depression, 0.08 (0.06) (Additional File 1).

Similarly, the annual mean OOPE (SD) of the participants was 2547 (2687) USD and the mean patient satisfaction score (SD) on a scale from 0 to 28 was 19.14 (3.01).

### HRQoL across sociodemographic and clinical characteristics

The mean (SD) values of the EQ-5D-5L index and the EQ-VAS scores were 0.39 (0.42) and 56.65 (21.71), respectively. The HRQoL of the study participants across different sociodemographic and clinical characteristics is presented in Table 1 (expanded table in Additional File). In the bivariate analysis, there was a significant difference in the mean EQ-5D-5L index score across age groups (*p* = 0.037), types of cancer (*p* = 0.026), cancer stages at diagnosis (*p* < 0.001), inpatient admissions (*p* = 0.012), OOPE categories (*p* < 0.001), and patient satisfaction (*p* = 0.018). Similarly, there was a significant difference in the mean EQ-VAS score across age groups (*p* = 0.008), religions (*p* = 0.007), types of cancer (*p* = 0.003) and cancer stages at diagnosis (*p* = 0.004). The correlation test showed that there was a weak negative correlation between OOPE and the EQ-5D-5L index (ρ= -0.225, *p* < 0.001), a weak positive correlation between patient satisfaction score and the EQ-5D-5L index (ρ = 0.147, *p* = 0.006), and a moderate positive correlation between the EQ-5D-5L score and the EQ-VAS (ρ = 0.491, *p* < 0.001).

**Table 1:** Mean EQ-5D-5L index and EQ-VAS scores across sociodemographic and clinical characteristics of the study participants

Characteristics	*N* (%) *n* = 353	EQ-5D-5L index score Mean (SD)	*p* value*	EQ-VAS score Mean (SD)	*p* value*
Age (years)					
20–39	48 (13.6)	0.51 (0.39)	**0.037**	61.94 (21.05)	**0.008**
40–59	158 (44.8)	0.40 (0.40)		59.55 (19.76)	
60 and above	147 (41.6)	0.35 (0.44)		51.85 (23.15)	
**Gender**					
Male	96 (27.2)	0.42 (0.38)	0.786	55.37 (21.68)	0.609
Female	257 (72.8)	0.38 (0.43)		57.12 (21.76)	
**Ethnicity**					
Hill Brahmin/Chhetri	106 (30.0)	0.44 (0.40)	0.402	58.19 (21.14)	0.270
Madhesi	42 (11.9)	0.44 (0.42)		60.21 (22.74)	
Janajati	163 (46.2)	0.37 (0.43)		56.11 (21.16)	
Others	42 (11.9)	0.34 (0.40)		51.51 (23.82)	
**Religion**					
Hindu	283 (80.2)	0.40 (0.42)	0.715	56.61 (21.71)	**0.007**
Buddhist	47 (13.3)	0.42 (0.38)		62.63 (18.31)	
Others	23 (6.5)	0.31 (0.45)		45.19 (24.50)	
**Residence**					
Urban	249 (70.5)	0.41 (0.43)	0.157	56.91 (22.53)	0.279
Rural	104 (29.5)	0.36 (0.40)		56.02 (19.76)	
**Education**					
No formal education	205 (58.1)	0.35 (0.43)	0.054	54.25 (22.16)	0.114
Basic education	78 (22.1)	0.48 (0.41)		60.94 (21.51)	
Secondary education and above	70 (19.8)	0.43 (0.37)		58.63 (19.92)	
**Type of family**					
Nuclear	134 (38.0)	0.44 (0.44)	0.050	55.89 (22.14)	0.679
Joint/Extended	219 (62.0)	0.37 (0.40)		57.10 (21.50)	
**Marital status**					
Currently married	300 (85.1)	0.42 (0.40)	0.074	57.24 (21.43)	0.440
Not currently married	53 (14.9)	0.26 (0.50)		53.05 (23.30)	
**Occupation**					
Not working and did not work in last 12 months	156 (44.2)	0.37 (0.44)	0.062	58.71 (22.86)	0.140
Employed	76 (21.5)	0.48 (0.37)		56.70 (21.67)	
Agriculture	66 (18.7)	0.33 (0.40)		53.24 (18.31)	
Others	55 (15.6)	0.45 (0.40)		56.80 (23.41)	
**Wealth quintile**					
Lowest	72 (20.4)	0.41 (0.40)	0.371	59.88 (20.07)	0.654
Lower	82 (23.2)	0.32 (0.44)		54.18 (23.35)	
Middle	46 (13.0)	0.50 (0.29)		57.97 (15.98)	
Higher	82 (23.2)	0.40 (0.42)		57.21 (22.24)	
Highest	71 (20.1)	0.39 (0.47)		54.31 (23.83)	
**Type of cancer**					
Lungs	89 (25.2)	0.29 (0.44)	**0.026**	50.93 (21.25)	**0.003**
Breast	82 (23.2)	0.48 (0.39)		63.70 (21.15)	
Cervical	92 (26.1)	0.42 (0.43)		59.73 (19.88)	
Stomach	57 (16.1)	0.35 (0.39)		52.68 (23.12)	
Oesophagus	33 (9.3)	0.46 (0.41)		52.41 (22.54)	
**Cancer staging**					
Stage I	55 (15.6)	0.50 (0.39)	**< 0.001**	62.70 (21.86)	**0.004**
Stage II	110 (31.2)	0.48 (0.35)		61.96 (18.33)	
Stage III	77 (21.8)	0.40 (0.41)		53.52 (19.33)	
Stage IV	87 (24.6)	0.21 (0.47)		52.65 (23.46)	
Not mentioned	24 (6.8)	0.40 (0.39)		44.78 (26.25)	
**Duration of treatment**					
Less than 6 months	211 (59.8)	0.41 (0.39)	0.958	56.53 (21.29)	0.627
6 months to 1 year	85 (24.1)	0.38 (0.42)		54.90 (23.34)	
Above one year	57 (16.1)	0.34 (0.51)		59.46 (21.32)	
**Treatment modality**					
Singular	239 (67.7)	0.42 (0.40)	0.156	56.80 (22.08)	0.848
Combination	114 (32.3)	0.34 (0.45)		56.31 (20.99)	
**Admission to inpatient care**					
No	110 (31.2)	0.47 (0.40)	**0.012**	58.88 (21.35)	0.082
Yes	243(68.8)	0.36 (0.42)		55.49 (21.86)	
**OOPE (quintiles)**					
Lowest	71 (20.1)	0.50 (0.45)	**< 0.001**	55.53 (26.47)	0.108
Lower	71 (20.1)	0.45 (0.38)		62.68 (17.16)	
Middle	70 (19.8)	0.37 (0.39)		55.64 (20.33)	
Higher	71 (20.1)	0.45 (0.35)		56.76 (20.11)	
Highest	70 (19.8)	0.20 (0.45)		51.78 (22.96)	
**Patient satisfaction**					
Dissatisfied	114 (32.3)	0.32 (0.42)	**0.018**	54.59 (21.42)	0.264
Satisfied	239 (67.7)	0.43 (0.41)		57.63 (21.84)	

*p values obtained from the Mann‒Whitney U test or Kruskal‒Wallis H test

### Association between OOPE and HRQoL

The output of the linear regression analysis with robust standard errors is presented in Table 2. In the Model I, an increase in log OOPE (β = -0.086; 95% CI: -0.132 to -0.040) was significantly associated with a lower EQ-5D-5L index score. This suggests that for every 1% increase in annual OOPE, the HRQoL decreases by 0.086 units, on average. Similarly, in the model II, individuals in the highest OOPE quintile had an average 0.224-point lower EQ-5D-5L index score compared to those in the lowest quintile (β = -0.224; 95% CI: - 0.369 to -  0.078).

Among the covariates in Model I, being currently not married (β = -0.149; 95% CI: -0.274 to -0.024) and having stage IV cancer during diagnosis (β = -0.212; 95% CI: -0.364 to -0.061) were significantly associated with a lower EQ-5D-5L index score than being currently married and having stage I cancer, respectively. Similarly, an increase in patient satisfaction score was significantly associated with a higher EQ-5D-5L index score (β = 0.015; 95% CI: 0.001 to 0.030). Age group, place of residence, education, type of family, occupation, type of cancer and inpatient admission in the past year were, however, not significantly associated with the EQ-5D-5L index score. The results were consistent across Model II, except that inpatient admission in the past year was significantly associated with the lower EQ-5D-5L index score.

**Table 2:** Determinants of HRQoL assessed by linear regression analysis with robust standard errors

Characteristics	Beta coefficients (95% CI): Model I	*p* value	Beta coefficients (95% CI): Model II	*p* value
Age group (ref: 20–39 years)				
40–59	-0.100 (-0.225 to 0.025)	0.117	-0.108 (-0.234 to 0.018)	0.093
60 and above	-0.087 (-0.230 to 0.057)	0.237	-0.107 (-0.253 to 0.040)	0.153
**Place of residence (ref: urban)**				
Rural	-0.025 (-0.117 to 0.067)	0.595	-0.032 (-0.127 to 0.062)	0.504
**Education (ref: no formal education)**				
Basic education	0.070 (-0.044 to 0.184)	0.226	0.094 (-0.020 to 0.207)	0.105
Secondary education and above	0.042 (-0.075 to 0.159)	0.480	0.030 (-0.088 to 0.149)	0.613
**Type of family (ref: nuclear)**				
Joint or extended	-0.038 (-0.126 to 0.050)	0.390	-0.038 (-0.127 to 0.052)	0.406
**Marital status (ref: currently married)**				
Not currently married (single, divorced, widowed)	-0.149 (-0.274 to -0.024)	**0.020**	-0.145 (-0.270 to -0.020)	**0.023**
**Occupation (ref: not currently working)**				
Employed	0.068 (-0.039 to 0.175)	0.210	0.044 (-0.064 to 0.152)	0.423
Agriculture	-0.072 (-0.185 to 0.041)	0.209	-0.074 (-0.190 to 0.041)	0.206
Others	0.005 (-0.120 to 0.130)	0.938	-0.004 (-0.129 to 0.121)	0.949
**Inpatient admission (ref: no)**				
Yes	-0.090 (-0.180 to 0.001)	0.051	-0.107 (-0.198 to -0.015)	**0.022**
**Type of cancer (ref: lung)**				
Breast	0.026 (-0.103 to 0.155)	0.689	0.004 (-0.127 to 0.135)	0.948
Cervical	0.009 (-0.125 to 0.143)	0.897	-0.009 (-0.143 to 0.125)	0.894
Stomach	0.005 (-0.127 to 0.137)	0.937	0.005 (-0.124 to 0.134)	0.937
Oesophagus	0.058 (-0.101 to 0.216)	0.475	0.045 (-0.121 to 0.210)	0.594
**Cancer stage during diagnosis (ref: stage I)**				
Stage II	-0.009 (-0.133 to 0.115)	0.887	-0.014 (-0.143 to 0.115)	0.832
Stage III	-0.008 (-0.147 to 0.132)	0.913	-0.004 (-0.147 to 0.138)	0.953
Stage IV	-0.212 (-0.364 to -0.061)	**0.006**	-0.216 (-0.367 to -0.066)	**0.005**
Not mentioned	-0.128 (-0.315 to 0.059)	0.178	-0.138 (-0.331 to 0.055)	0.160
**Patient satisfaction score**	0.015 (0.001 to 0.030)	**0.034**	0.015 (0.001 to 0.030)	**0.034**
**Log of annual OOPE**	-0.086 (-0.132 to -0.040)	**< 0.001**	**-**	**-**
**OOPE categories (ref: lowest)**	-	**-**		
Lower			-0.002 (-0.131 to 0.128)	0.981
Middle			-0.087 (-0.227 to 0.052)	0.219
Higher			-0.015 (-0.147 to 0.117)	0.826
Highest			-0.224 (-0.369 to -0.078)	**0.003**

**Fig. 2:**
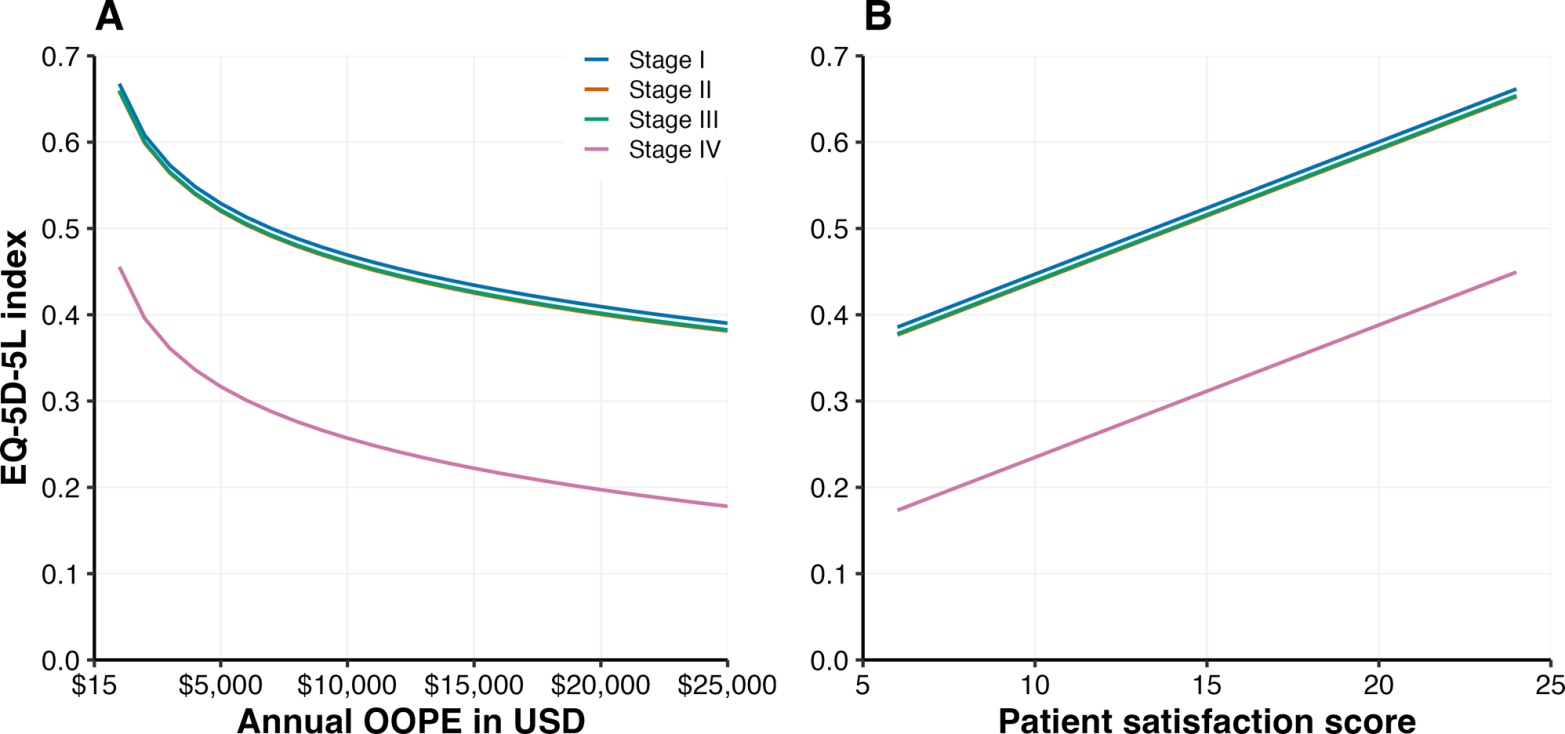
Predicted EQ-5D-5L index score by OOPE and patient satisfaction score across cancer stages Panel (**A**) shows the relationship between annual OOPE (in USD) and the predicted EQ-5D-5L index score for patients at different cancer stages, Panel (**B**) presents the predicted EQ-5D-5L index score as a function of patient satisfaction scores, showing a positive association across all cancer stages

The index scores predicted by OOPE and patient satisfaction across different cancer stages are shown in Fig. 2.

## Discussion

We found a moderate HRQoL among patients undergoing cancer treatment in Nepal with > 90% reporting problems related to pain/discomfort and anxiety/depression. Our study identified OOPE, patient satisfaction, advanced cancer stage, and marital status as factors associated with HRQoL.

Our study revealed a negative association between OOPE and HRQoL suggesting that financial burden adversely affect quality of life among patients with cancer. In Nepal, OOPE is the dominant source of financing health care, including cancer treatment, forcing patients to compromise with their necessities, pushing families into poverty traps, and affecting their psychological health [28, 40]. It is therefore necessary to address the financial implications of cancer treatment in Nepal by establishing a strong social health protection mechanism. This will require defining and integrating priority cancer interventions into the country’s health benefit packages (basic health services and/or health insurance) and linking them with service delivery platforms and financing mechanisms. Existing fragmented schemes such as treatment subsidies from federal and provincial governments, monthly cash allowances for cancer treatment, health insurance, and those for formal sector workers (employees provident fund and social security fund) should be either merged or harmonized to gain more efficiency and share financial risk across the population. Studies done elsewhere have reported mixed effects regarding the association between OOPE and HRQoL. A study on patients with early-stage lung cancer in China revealed no association [41], whereas another study from Finland involving breast, prostate, and colorectal cancer participants showed a negative association between OOPE and HRQoL [23].

We found a moderate EQ-5D-5L index score (0.39, stage I: 0.50, stage IV: 0.21) in our study participants while having stage IV cancer during diagnosis was significantly associated with a lower HRQoL compared to Stage I. While the reasons for low HRQoL among our study population need to be explored, we argue that it depicts the state of cancer care in Nepal and patients’ realization of their health and well-being while experiencing cancer. Studies from India using the same EQ-5D-5L value set have reported higher index scores. One study among breast cancer participants reported a mean score of 0.60 (stage I: 0.66 and stage IV: 0.56) [42] while another study, which included patients with various types of cancer, found a mean score of 0.63 (stage I: 0.64 and stage IV: 0.52) [43]. Additionally, another study focusing on breast cancer reported EQ-5D-5L index scores exceeding 0.80 across all stages of cancer [44]. The higher index scores among Indian participants could be partly explained by better health insurance coverage (introduction of schemes such as Ayushman Bharat) [45] and improved access to cancer treatment [46]. The lower HRQoL at advanced stage might be due to transition to metastatic disease with patients experiencing a higher burden of physical symptoms including pain and fatigue coupled with psychological distress. Studies from India [43, 44] and elsewhere [47–51] also reported a negative association between advanced cancer stages and HRQoL, while a study from India reported no association [42]. With existing evidence suggesting diagnostic delays among cancer participants in Nepal [52], our findings emphasize the importance of early detection, diagnosis, and management of cancer for improving the HRQoL of patients.

In our study, patient satisfaction score was positively associated with HRQoL, indicating that a patient’s experience with health care services help to positively shape their perceptions regarding their quality of life. Previous studies [53–55] also have shown a positive association between patient satisfaction and HRQoL, indicating the need to improve patients’ experience of care and build trust between patients and the health care system.

### Strengths and limitations

While we acknowledge the use of other HRQoL instruments among this population [56–59] and the use of EQ-5D among other population [34–37, 60–63], application of EQ-5D-5L has not been previously documented in patients with cancer in Nepal. It is relatively easier to use and widely applicable in economic evaluation. However, the study has several limitations including its cross-sectional design, which does not compare HRQoL across different time points. There also might have been recall bias in reporting OOPE. Despite limitations, this study offers evidence on the utility values, which could be used for economic evaluation, and identifies factors associated with HRQoL, which could guide policy makers and clinicians in health-care decision making.

## Conclusions

The study revealed a moderate HRQoL among patients undergoing cancer treatment in Nepal, with pain/discomfort bearing the greatest disability weight. Higher OOPE along with stage IV cancer at diagnosis and currently not married status were significantly associated with a lower HRQoL, whereas a higher patient satisfaction score was significantly associated with a higher HRQoL. These findings suggest a need to focus on reducing the financial burden among patients, investing in early diagnosis and management, and improving patient satisfaction at the health facility level to improve HRQoL among patients receiving cancer treatment. We recommend longitudinal and qualitative studies to understand the impact of OOPE on HRQoL among patients with cancer including those who have abandoned treatment due to financial barrier.

## Electronic supplementary material

Below is the link to the electronic supplementary material.

Supplementary Material 1

## Data Availability

All the data generated during this study are included in the manuscript and the additional file 1.

## Abbreviations

BCH: Bhaktapur Cancer Hospital
BPKMCH: BP Koirala Memorial Cancer Hospital
CI: Confidence interval
DALYs: Disability-adjusted life years
EQ-5D-5L: European Quality of Life 5 dimensions
EQ-VAS: European Quality of Life Visual Analogue Scale
HICs: High-income countries
HRQoL: Health-related quality of life
LLMICs: Low and lower- middleincome countries
NPR: Nepalese rupees
OOPE: Out-of-pocket expenditure
SAPS: Short Assessment of Patient Satisfaction
SD: Standard deviation
USD: United States Dollar
