# Supplementary material for: Association between out-of-pocket expenditure and health-related quality of life among patients receiving cancer treatment: a cross-sectional study from Nepal
Source: Health Qual Life Outcomes. 2025 Jul 15;23:73. doi: 10.1186/s12955-025-02404-9 (PMC12261754; doi:10.1186/s12955-025-02404-9)
Supplement: Supplementary file 1 — Supplementary Material 1 [file 12955_2025_2404_MOESM1_ESM.docx]

**Supplementary Tables and Figure**

**Supplementary Tables**

S1 Table Study variables

S2 Table Mean disability weight of five dimensions of EQ-5D-5L

S3Table Socio-demographic and treatment-related characteristics of the study participants along with mean EQ-5D-5L and EQ-VAS scores (Expanded table)

**Supplementary Figure**

Supplementary Fig. 1 Distribution of EQ-5D-5L index scores

**S1 Table Study variables**

| **S.N.** | **Variables** | **Categories of variables** |
| --- | --- | --- |
|  | Dependent variable |  |
| 1 | Health-related quality of life | Obtained from 5-item EQ-5D-5L. Converted to utility index using country value set |
|  | Independent variables |  |
|  | Socio-demographic characteristics |  |
| 1 | Age | In years, categorized as 20-39 years, 40-59 years, and 60 and above |
| 2 | Gender | female, male |
| 3 | Ethnicity | Hill Brahmin/Chhetri, Janajati, Madheshi, Dalit, Muslim, and others. Dalits and Muslim were grouped as others during analysis. Adapted as per Nepal’s Health Management Information System |
| 4 | Religion | Hindu, Buddhist, others. Others include Christian, Muslim and Kirat |
| 5 | Province | Koshi, Madhesh, Bagmati, Gandaki, Lumbini, Karnali, and Sudurpashchim. For analysis, Karnali and Sudurpaschchim were grouped together due to low sample size within Karnali sub-group. |
| 6 | Residence | Urban, Rural |
| 7 | Education | no formal education, basic education, secondary education and above |
| 8 | Type of family | Nuclear and joint/extended |
| 9 | Family size | up to five, greater than five |
| 10 | Marital status | Currently married, not currently in union (never married, divorced, separated, widowed) |
| 11 | Occupation | not working and did not work in the last 12 months, agriculture, employed (sales and service, professional/technical/managerial, unskilled manual, skilled manual, clerical) and others (including homemaker) |
| 12 | Number of economically active family members | None, one, greater than one |
| 13 | Wealth quintile | lowest, lower, middle, higher and highest. based on annual household consumption. |
|  | Treatment related variables |  |
| 1 | Type of cancer | Lung, Cervical, Breast, Stomach, and Oesophagus |
| 2 | Duration of diagnosis | categorized as less than six months, 6 months to one year, above one year |
| 3 | Staging of cancer | stage I, stage II, stage III, stage IV, not mentioned |
| 4 | Duration of treatment | categorized as less than six months, 6 months to one year, above one year |
| 5 | Treatment modality | singular, combination |
| 6 | Visited private health facility before coming to the study hospital | yes, no |
| 7 | Presence of other chronic diseases | yes, no |
| 8 | Study site | BP Koirala Memorial Cancer Hospital, Bhaktapur Cancer Hospital |
| 9 | Travelling distance to the study site | less than one hour, 1 hour to 6 hours, greater than 6 hours |
| 10 | Admission to inpatient care last year (past 365 days) | yes, no |
|  | Cost related variable |  |
| 1 | Annual out-of-pocket payment expenditure | Sum of direct medical and direct non-medical costs in last one year. Currency reported in US Dollars. Log transformation done to adjust the cost variation. |
|  | Patient satisfaction related variable |  |
| 1 | Patient satisfaction | Sum of the seven-item short assessment of patient satisfaction score (ranges from 0 to 28), categorized as SAPS scores are interpreted as very dissatisfied (0 to10), dissatisfied (11 to18), satisfied (19 to 26) or very satisfied (27 to 28). |

**S2 Table Mean disability weight of five dimensions of EQ-5D-5L**

| **Dimension of EQ-5D-5L** | **Mean disability weight (95% CI)** | **Standard deviation** |
| --- | --- | --- |
| Mobility | 0.09 (0.08-0.10) | 0.10 |
| Self care | 0.13 (0.12-0.15) | 0.13 |
| Usual activities | 0.13 (0.12-0.14) | 0.11 |
| Pain/Discomfort | 0.17 (0.15-0.18) | 0.15 |
| Anxiety/Depression | 0.08 (0.07-0.08) | 0.06 |
| Total disability weight | 0.61 (0.56-0.65) | 0.42 |
| EQ-5D-5L index score (1-total disability weight) | 0.39 (0.35-0.44) | 0.42 |

**S3Table Socio-demographic and treatment-related** **characteristics of the study participants along with mean EQ-5D-5L and EQ-VAS scores (Expanded table)**

| **Characteristics** | **N (%)**  n=353 | **EQ-5D-5L Utility score**  Mean (SD) | **p-value** | **EQ-VAS score**  Mean (SD) | **p-value** |
| --- | --- | --- | --- | --- | --- |
| **Age (years)** |  |  |  |  |  |
| 20-39 | 48 (13.6) | 0.51 (0.39) | **0.037** | 61.94 (21.05) | **0.008** |
| 40-59 | 158 (44.8) | 0.40 (0.40) |  | 59.55 (19.76) |  |
| 60 and above | 147 (41.6) | 0.35 (0.44) |  | 51.85 (23.15) |  |
| **Gender** |  |  |  |  |  |
| Male | 96 (27.2) | 0.42 (0.38) | 0.786 | 55.37 (21.68) | 0.609 |
| Female | 257 (72.8) | 0.38 (0.43) |  | 57.12 (21.76) |  |
| **Ethnicity** |  |  |  |  |  |
| Hill Brahmin/Chhetri | 106 (30.0) | 0.44 (0.40) | 0.402 | 58.19 (21.14) | 0.270 |
| Madhesi | 42 (11.9) | 0.44 (0.42) |  | 60.21 (22.74) |  |
| Janajati | 163 (46.2) | 0.37 (0.43) |  | 56.11 (21.16) |  |
| Others | 42 (11.9) | 0.34 (0.40) |  | 51.51 (23.82) |  |
| **Religion** |  |  |  |  |  |
| Hindu | 283 (80.2) | 0.40 (0.42) | 0.715 | 56.61 (21.71) | **0.007** |
| Buddhist | 47 (13.3) | 0.42 (0.38) |  | 62.63 (18.31) |  |
| Others | 23 (6.5) | 0.31 (0.45) |  | 45.19 (24.50) |  |
| **Province** |  |  |  |  |  |
| Koshi | 46 (13.0) | 0.34 (0.40) | 0.298 | 50.82 (22.41) | 0.059 |
| Madhesh | 32 (9.1) | 0.28 (0.50) |  | 54.14 (23.45) |  |
| Bagmati | 126 (35.7) | 0.45 (0.40) |  | 60.53 (21.84) |  |
| Gandaki | 55 (15.6) | 0.41 (0.46) |  | 55.39 (19.81) |  |
| Lumbini | 57 (16.1) | 0.34 (0.39) |  | 56.36 (23.04) |  |
| Karnali | 12 (3.4) | 0.41 (0.46) |  | 40.63 (24.92) |  |
| Sudurpashchim | 25 (7.1) | 0.46 (0.32) |  | 61.30 (12.99) |  |
| **Residence** |  |  |  |  |  |
| Urban | 249 (70.5) | 0.41 (0.43) | 0.157 | 56.91 (22.53) | 0.279 |
| Rural | 104 (29.5) | 0.36 (0.40) |  | 56.02 (19.76) |  |
| **Education** |  |  |  |  |  |
| No formal education | 205 (58.1) | 0.35 (0.43) | 0.054 | 54.25 (22.16) | 0.114 |
| Basic education | 78 (22.1) | 0.48 (0.41) |  | 60.94 (21.51) |  |
| Secondary education and above | 70 (19.8) | 0.43 (0.37) |  | 58.63 (19.92) |  |
| **Type of family** |  |  |  |  |  |
| Nuclear | 134 (38.0) | 0.44 (0.44) | 0.050 | 55.89 (22.14) | 0.679 |
| Joint/Extended | 219 (62.0) | 0.37 (0.40) |  | 57.10 (21.50) |  |
| **Marital status** |  |  |  |  |  |
| Married | 300 (85.1) | 0.42 (0.40) | 0.074 | 57.24 (21.43) | 0.440 |
| Not currently in union | 53 (14.9) | 0.26 (0.50) |  | 53.05 (23.30) |  |
| **Study site** |  |  |  |  |  |
| BPKMCH | 186 (52.7) | 0.37 (0.42) | 0.253 | 55.49 (21.96) | 0.134 |
| BCH | 167 (47.3) | 0.42 (0.41) |  | 58.58 (21.25) |  |
| **Travel time** |  |  |  |  |  |
| Less than one hour | 56 (15.9) | 0.41 (0.46) | 0.213 | 57.42 (22.49) | 0.250 |
| One hour to six hours | 157 (44.5) | 0.42 (0.42) |  | 58.26 (21.59) |  |
| More than 6 hours | 140 (39.7) | 0.36 (0.40) |  | 54.64 (21.62) |  |
| **Family size** |  |  |  |  |  |
| Up to 5 | 191 (54.1) | 0.42 (0.42) | 0.140 | 56.56 (21.59) | 0.493 |
| Greater than 5 | 162 (45.9) | 0.36 (0.41) |  | 56.75 (21.93) |  |
| **Occupation** |  |  |  |  |  |
| Not working and did not work in last 12 months | 156 (44.2) | 0.37 (0.44) | 0.062 | 58.71 (22.86) | 0.140 |
| Employed | 76 (21.5) | 0.48 (0.37) |  | 56.70 (21.67) |  |
| Agriculture | 66 (18.7) | 0.33 (0.40) |  | 53.24 (18.31) |  |
| Others | 55 (15.6) | 0.45 (0.40) |  | 56.80 (23.41) |  |
| **Number of economically active family members** |  |  |  |  |  |
| None | 18 (5.1) | 0.48 (0.40) | 0.570 | 61.00 (31.13) | 0.517 |
| One | 174 (49.3) | 0.38 (0.41) |  | 55.20 (21.15) |  |
| Two or more | 161(45.6) | 0.40 (0.42) |  | 57.69 (21.12) |  |
| **Wealth quintile** |  |  |  |  |  |
| Lowest | 72 (20.4) | 0.41 (0.40) | 0.371 | 59.88 (20.07) | 0.654 |
| Lower | 82 (23.2) | 0.32 (0.44) |  | 54.18 (23.35) |  |
| Middle | 46 (13.0) | 0.50 (0.29) |  | 57.97 (15.98) |  |
| Higher | 82 (23.2) | 0.40 (0.42) |  | 57.21 (22.24) |  |
| Highest | 71 (20.1) | 0.39 (0.47) |  | 54.31 (23.83) |  |
| **Member of NHIP** |  |  |  |  |  |
| No | 160 (45.3) | 0.41 (0.41) | 0.505 | 56.38 (22.84) | 0.940 |
| Yes | 193 (54.7) | 0.38 (0.42) |  | 56.87 (20.79) |  |
| **Member of any health protection scheme** |  |  |  |  |  |
| None | 146 (41.4) | 0.40 (0.41) | 0.873 | 56.36 (22.99) | 0.823 |
| At least one | 207 (58.6) | 0.39 (0.42) |  | 56.86 (20.78) |  |
| **Received chronic disease-related subsidy** |  |  |  |  |  |
| None | 37 (10.5) | 0.39 (0.43) | 0.923 | 52.85 (22.61) | 0.256 |
| At least one source | 316 (89.5) | 0.39 (0.43) |  | 57.15 (21.59) |  |
| **Type of cancer** |  |  |  |  |  |
| Lungs | 89 (25.2) | 0.29 (0.44) | **0.026** | 50.93 (21.25) | **0.003** |
| Breast | 82 (23.2) | 0.48 (0.39) |  | 63.70 (21.15) |  |
| Cervical | 92 (26.1) | 0.42 (0.43) |  | 59.73 (19.88) |  |
| Stomach | 57 (16.1) | 0.35 (0.39) |  | 52.68 (23.12) |  |
| Oesophagus | 33 (9.3) | 0.46 (0.41) |  | 52.41 (22.54) |  |
| **Duration of diagnosis** |  |  |  |  |  |
| < 6 months | 199 (56.4) | 0.40 (0.39) | 0.977 | 56.75 (20.59) | 0.682 |
| 6 months to 1 year | 96 (27.2) | 0.40 (0.41) |  | 54.55 (24.91) |  |
| > one year | 58 (16.4) | 0.35 (0.50) |  | 59.27 (20.91) |  |
| **Cancer staging** |  |  |  |  |  |
| Stage I | 55 (15.6) | 0.50 (0.39) | **<0.001** | 62.70 (21.86) | **0.004** |
| Stage II | 110 (31.2) | 0.48 (0.35) |  | 61.96 (18.33) |  |
| Stage III | 77 (21.8) | 0.40 (0.41) |  | 53.52 (19.33) |  |
| Stage IV | 87 (24.6) | 0.21 (0.47) |  | 52.65 (23.46) |  |
| Not mentioned | 24 (6.8) | 0.40 (0.39) |  | 44.78 (26.25) |  |
| **Duration of treatment** |  |  |  |  |  |
| Less than 6 months | 211 (59.8) | 0.41 (0.39) | 0.958 | 56.53 (21.29) | 0.627 |
| 6 months to 1 year | 85 (24.1) | 0.38 (0.42) |  | 54.90 (23.34) |  |
| Above one year | 57 (16.1) | 0.34 (0.51) |  | 59.46 (21.32) |  |
| **Treatment modality** |  |  |  |  |  |
| Singular | 239 (67.7) | 0.42 (0.40) | 0.156 | 56.80 (22.08) | 0.848 |
| Combination | 114 (32.3) | 0.34 (0.45) |  | 56.31 (20.99) |  |
| **Visited private health facility** |  |  |  |  |  |
| No | 135 (38.2) | 0.42 (0.42) | 0.339 | 54.61 (24.51) | 0.658 |
| Yes | 218(61.8) | 0.38 (0.41) |  | 57.78 (19.97) |  |
| **Presence of other chronic diseases** |  |  |  |  |  |
| No | 235 (66.6) | 0.41 (0.39) | 0.689 | 56.38 (21.93) | 0.766 |
| Yes | 118 (33.4) | 0.36 (0.47) |  | 57.21 (21.35) |  |
| **Admission to inpatient care** |  |  |  |  |  |
| No | 110 (31.2) | 0.47 (0.40) | **0.012** | 58.88 (21.35) | 0.082 |
| Yes | 243(68.8) | 0.36 (0.42) |  | 55.49 (21.86) |  |
| **OOPE (quartiles)** |  |  |  |  |  |
| Q1 | 71 (20.1) | 0.50 (0.45) | **<0.001** | 55.53 (26.47) | 0.108 |
| Q2 | 71 (20.1) | 0.45 (0.38) |  | 62.68 (17.16) |  |
| Q3 | 70 (19.8) | 0.37 (0.39) |  | 55.64 (20.33) |  |
| Q4 | 71 (20.1) | 0.45 (0.35) |  | 56.76 (20.11) |  |
| Q5 | 70 (19.8) | 0.20 (0.45) |  | 51.78 (22.96) |  |
| **Patient satisfaction** |  |  |  |  |  |
| Dissatisfied | 114 (32.29) | 0.32 (0.42) | **0.018** | 54.59 (21.42) | 0.264 |
| Satisfied | 239 (67.71) | 0.43 (0.41) |  | 57.63 (21.84) |  |


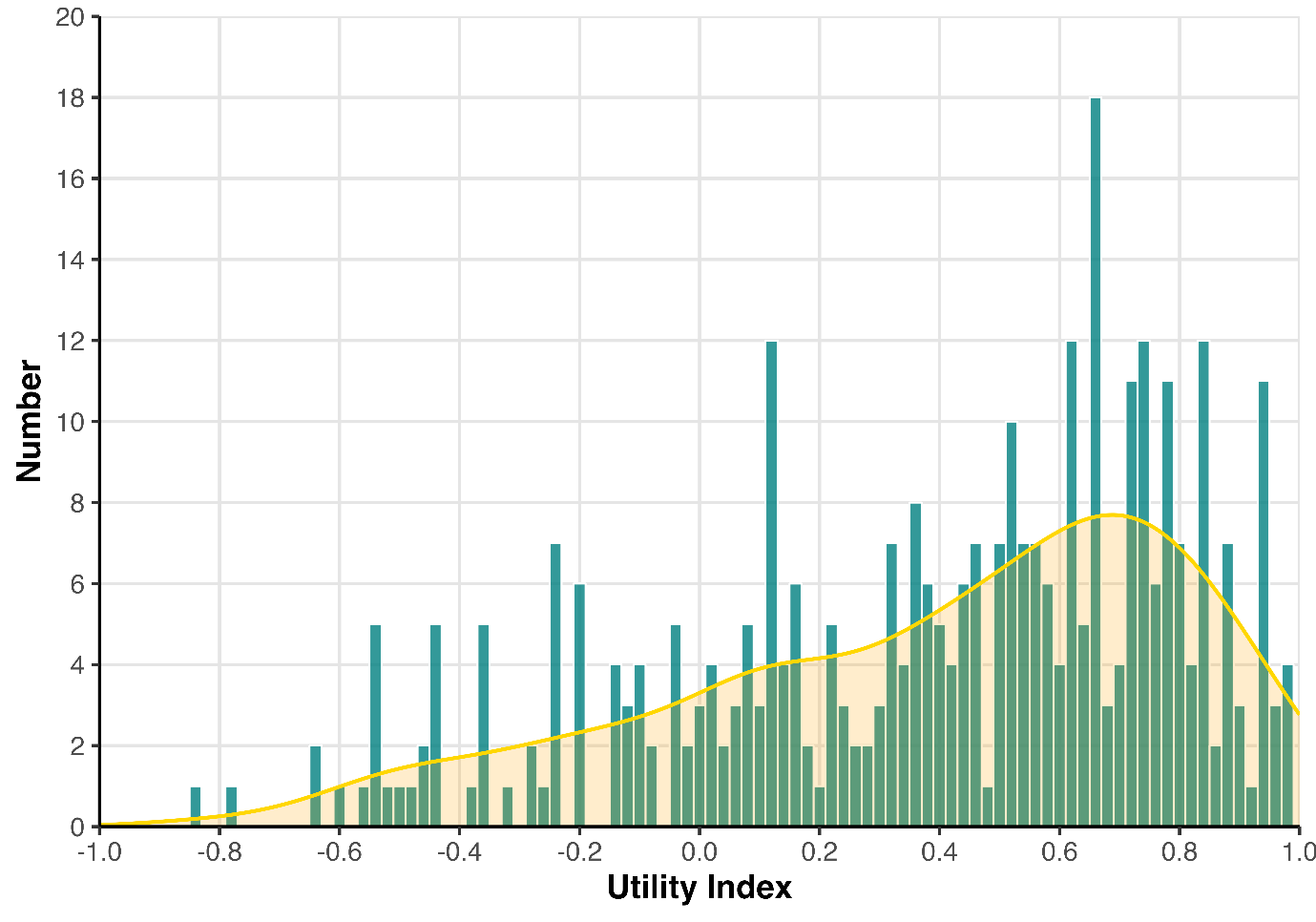


**Supplementary Fig. 1 Distribution of EQ-5D-5L index scores**
